# Bioactive Three-Dimensional Chitosan-Based Scaffolds Modified with Poly(dopamine)/CBD@Pt/Au/PVP Nanoparticles as Potential NGCs Applicable in Nervous Tissue Regeneration—Preparation and Characterization

**DOI:** 10.3390/molecules29225376

**Published:** 2024-11-14

**Authors:** Aleksandra Sierakowska-Byczek, Aleksandra Gałuszka, Łukasz Janus, Julia Radwan-Pragłowska

**Affiliations:** Department of Biotechnology and Physical Chemistry, Faculty of Chemical Engineering and Technology, Cracow University of Technology, Warszawska 24 Street, 31-155 Cracow, Poland; aleksandra.sierakowska-byczek@doktorant.pk.edu.pl (A.S.-B.); galuszka2000@wp.pl (A.G.); lukasz.janus@pk.edu.pl (Ł.J.)

**Keywords:** functionalized nanomaterials, compositions, biopolymers, biomaterials, tissue engineering

## Abstract

Tissue engineering of nervous tissue is a promising direction in the treatment of neurological diseases such as spinal cord injuries or neuropathies. Thanks to technological progress and scientific achievements; the use of cells; artificial scaffolds; and growth factors are becoming increasingly common. Despite challenges such as the complex structure of this tissue, regenerative medicine appears as a promising future approach to improve the quality of life of patients with nervous injuries. Until now; most functional biomaterials used for this purpose were based on decellularized extra cellular matrix (ECM) or nanofibrous materials, whereas current clinically verified ones in most cases do not exhibit bioactivity or the possibility for external stimulation. The aim of this research was to develop a new type of bioactive, chitosan-based 3D materials applicable as nerve guide conduits (NGCs) modified with poly(dopamine), Au/Pt coated with PVP nanoparticles, and cannabidiol. The NGCs were prepared under microwave-assisted conditions and their chemical structure was studied using the FT-IR method. Next, this study will discuss novel biomaterials for morphology and swelling abilities as well as susceptibility to biodegradation in the presence of collagenase and lysozyme. Finally, their potential in the field of nervous tissue engineering has been verified via a cytotoxicity study using the 1321N1 human astrocytoma cell line, which confirmed their biocompatibility in direct contact studies.

## 1. Introduction

Tissue engineering of nervous tissue is a promising direction for the treatment of neurological disorders such as spinal cord injuries or neuropathies. Thanks to technological progress and scientific achievements, the use of stem cells, artificial scaffolds, and growth factors is becoming increasingly common. The development of personalized therapies and commercial potential further emphasizes the importance of this field. Despite challenges such as the complex structure of nervous tissue and the need for long-term clinical trials, tissue engineering of nervous tissue is emerging as a future way to improve the quality of life of patients with neurological injuries [1,2,3,4].

Key strategies for tissue engineering of nervous tissue include the use of biodegradable artificial scaffolds, stem cells, growth factors, and 3D printing technology. Polymers play a key role in tissue engineering due to their versatile applications and functional importance. Their versatile applications include various functions, which makes them extremely important in the process of tissue regeneration. Polymers can be formed into three-dimensional matrices, providing a foundation for cells to adhere, grow, and differentiate. These artificial matrices provide an appropriate environment for cells, allowing them to develop in a controlled manner. Second, polymers are used as drug delivery vehicles. They can be designed to release active substances at a controlled rate and targeted location. Third, polymer biomaterials provide a scaffold that supports the tissue regeneration process. They are usually degradable, meaning that they are absorbed by the body over time, leaving behind reconstructed tissue [5,6,7]. Fourth, polymers are used to create artificial organs and tissues, such as artificial blood vessels, bones, skin, or corneas. These structures can be implanted in the human body to replace or support the function of damaged organs or tissues. In each of these applications, it is important to select the right polymers, taking into account their biocompatibility, degradability, mechanical properties, and ability to interact with cells. As a result, tissue engineering is becoming an increasingly promising field in regenerative medicine [8,9,10,11].

So far, significant developments regarding skin, cartilage and bone regeneration have been achieved, resulting in the obtainment of constructs mimicking healthy tissue. However, there are still many areas which are left behind and lack functional, bioactive solutions in terms of their restoration. One of the most overlooked areas is nervous tissue, which is a key part of the nervous system, enabling communication, control of bodily functions, and responses to external stimuli. It is made up of a variety of components, including neurons (nerve cells), axons, synaptic endings, and glial cells. Neurons are the basic functional units of nervous tissue [12,13,14,15,16,17,18,19].

The strategy for treating nerve injuries depends on the type of injury. Currently, autologous nerve transplantation is considered the gold standard in the treatment of tissue and organ defects. The donor site is usually a functionally less important segment. However, it should be noted that this approach has significant drawbacks, including limited biological material that can be harvested for nerve supply, high morbidity at the donor site, and the need for more than one surgical procedure. 

Other options include allogeneic nerve grafts, which consist of material harvested from a cadaver or a donor of the same species, and xenogeneic nerve grafts, whose harvested material comes from a different species. Although allografts have viable Schwann cells (SCs), they require immunosuppressive therapy for a longer period, from 18 to 24 months, which is a major disadvantage that increases the risk of opportunistic infections and malignancies. Allografts are used in cases where autograft material is unusable or insufficient. There are several models of nerve repair developed by neural tissue engineering. They consist of a combination of 3 elements: stimuli, which can be electrical, biochemical, and topographic, cells, and biomaterials, which are used to create axonal conduits, hydrogels and fibers [20,21,22,23,24,25].

Nerve guidance conduits (NGCs), when used as a standalone therapy, show limited efficacy because the regeneration process requires an environment with specific pro-regenerative stimuli and mechanical support.

In this respect, cellular systems promote the creation of a microenvironment favorable for axonal regeneration and can optimize the regeneration process in lesions with large gaps. Therefore, the introduction of in vitro cultured cells and tissues into artificial nerve conduits and their combination with other therapies has been the subject of many studies in the field of tissue engineering and regenerative medicine. The most promising raw materials for NGC preparation include biopolymers such as collagen, fibrin, hyaluronic acid, and chitosan—a bioactive chitin derivative which exhibits numerous favorable biological properties, mainly due to the free amino groups present in its structure [1,2,3,4,5,20]. However, NGCs of the new generation should not only exhibit biocompatibility and mechanical support, but also enable cell proliferation and maturation stimulation via biochemical or physical factors. For this reason, conductive polymers such as nanoparticles constitute very promising additives during the manufacturing process. Recently, significant attention in the field of nervous tissue engineering has been given to such compounds like poly(dopamine) [26,27,28,29,30,31,32,33,34,35,36] and cannabidiol [37,38,39,40,41], since they have been confirmed for their bioactivity and interactions with neural cells. Thus, they constitute a promising modifying agent that may positively influence nerve regeneration [42].

The aim of the following research was to obtain and investigate the physicochemical and biological properties of new biomaterials based on chitosan of non-animal origin to determine their potential in the reconstruction of nervous tissue. This goal was achieved by synthesizing an aerogel under microwave-assisted conditions, which was then lyophilized to a three-dimensional scaffold structure. Chitosan of fungal origin, l-Glutamic acid, l-Aspartic acid, and propylene glycol were used for chemical crosslinking, whereas as active substances poly(dopamine) nanoparticles and cannabidiol were used to increase the polymeric scaffold in nervous tissue regeneration. To verify their cytocompatibility, cytotoxicity studies were carried out on astrocytes (1321N1 cell line) to examine cellular behavior as a result of direct astrocyte-biomaterial interactions.

## 2. Results 

**Figure 1:**
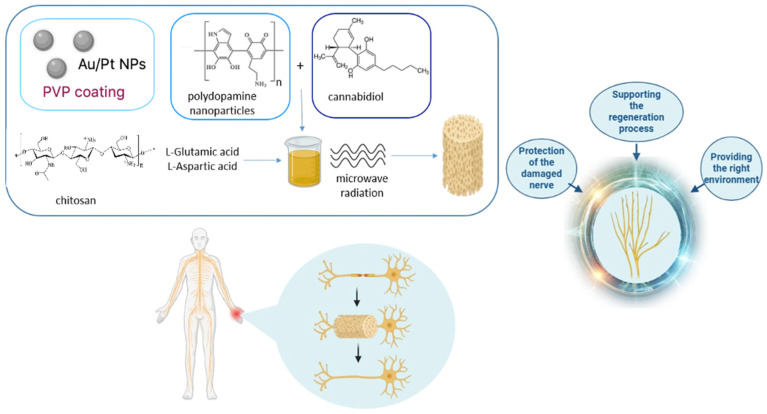
General biomaterial preparation scheme.

Figure 1 presents the general scheme of novel biomaterials obtainment which were prepared under microwave-assisted conditions.

### 2.1. Nanoparticles Morphology Study

**Figure 2:**
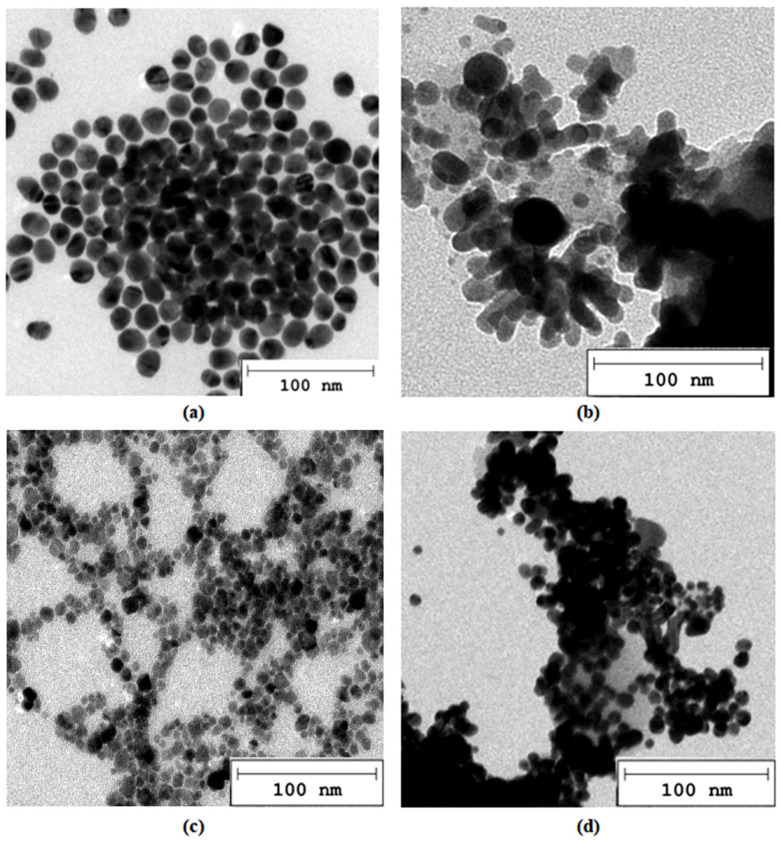
TEM microphotographs (**a**) Au nanoparticles; (**b**) Au/PVP nanoparticles; (**c**) Pt nanoparticles; (**d**) Pt/PVP nanoparticles.

The first stage in preparing novel NGCs with conductive properties to enable electrostimulation and accelerate regeneration involved obtaining stable metallic nanoparticles that retained their conductivity. Figure 2 shows the morphology of unmodified Au (Figure 2a) and Pt (Figure 2c) nanoparticles, as well as nanoparticles coated with the conductive, non-toxic polymer PVP (Figure 2b,d). The average diameter of the nanoparticles in each case was approximately 20 nm, suggesting they should not induce nephrotoxicity, as they are unlikely to accumulate in the kidneys or other organs. Both types of nanoparticles exhibit a round, homogeneous shape (Figure 2a,c). The addition of PVP resulted in a superficial modification, forming a conductive coating on the nanoparticles. This coating not only enhances the electrical properties of the nano-additives, but also potentially improves their integration with the polymer matrix [43].

### 2.2. Fourier Transform Infrared Spectroscopy (FT-IR)

**Figure 3:**
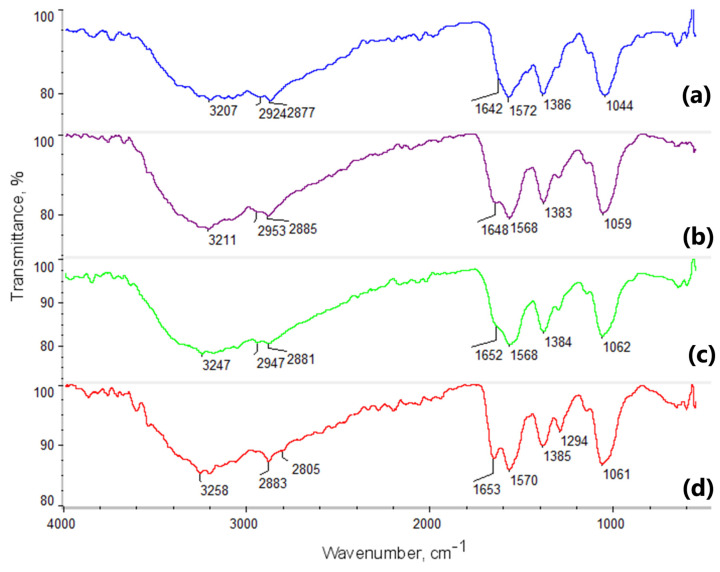
FTIR spectra of chitosan biomaterials modified with poly(dopamine)@Au/PVP NPs: (**a**) sample **1**; (**b**) sample **2**; (**c**) sample **3**; (**d**) sample **4**.

**Figure 4:**
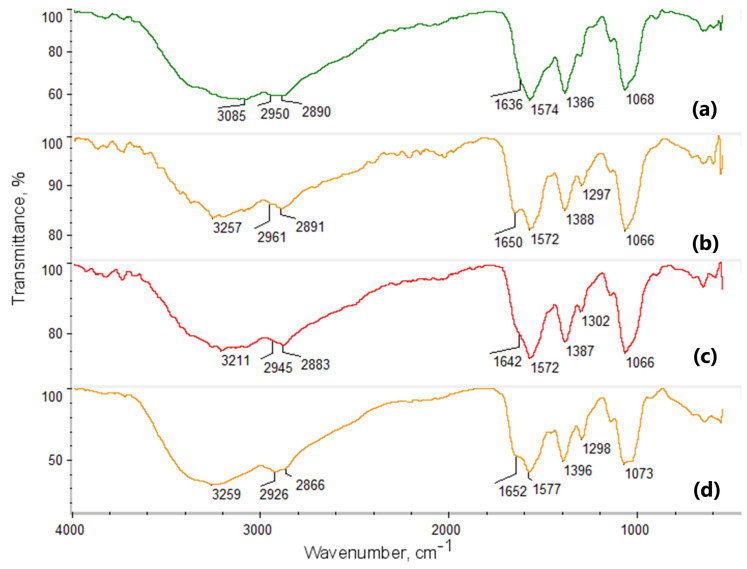
FTIR spectra of chitosan biomaterials modified with CBD/Au/PVP NPs: (**a**) sample **5**; (**b**) sample **6**; (**c**) sample **7**; (**d**) sample **8**.

**Figure 5:**
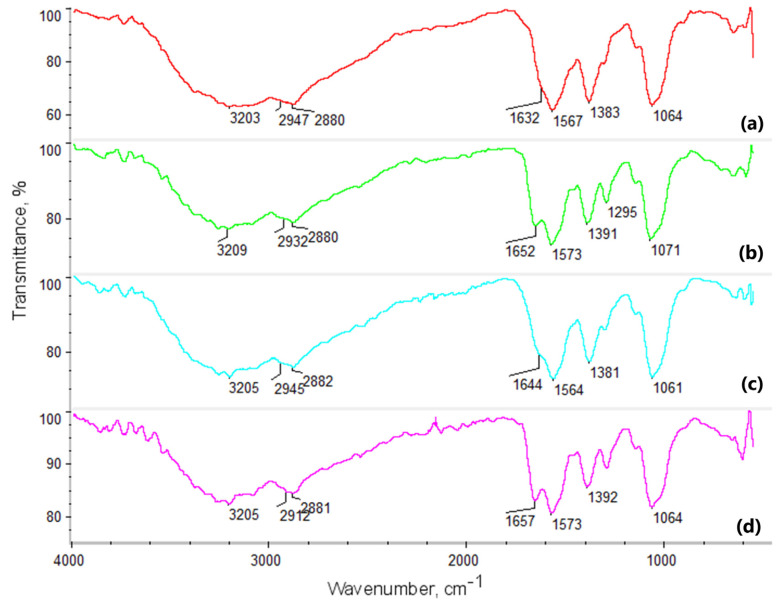
FTIR spectra of chitosan biomaterials modified with poly(dopamine)@Pt/PVP NPs: (**a**) sample **9**; (**b**) sample **10**; (**c**) sample **11**; (**d**) sample **12**.

Intermolecular interactions play a crucial role in the design of novel biomaterials, influencing numerous physicochemical and biological properties. Consequently, any biomaterial intended for biomedical applications should undergo structural analysis before use. Figure 3, Figure 4 and Figure 5 present the FT-IR analysis of the newly developed potential NGCs.

The FT-IR spectra reveal characteristic absorption bands associated with chitosan, glutamic acid, aspartic acid, cannabidiol, and poly(dopamine). Identifying various functional groups and monitoring structural changes in these compounds provides a deeper understanding of their chemical properties and interactions under different experimental conditions.

Key regions of the FT-IR spectrum for chitosan (Figure 3) include bands corresponding to O-H and N-H stretching, appearing in the 3200–3500 cm^−1^ range, and bands in the 2800–2900 cm^−1^ range (2881, 2882, and 2886 cm^−1^) related to C-H stretching in the aliphatic chains of chitosan. Characteristic bands for amide groups appear around 1650 cm^−1^, corresponding to C=O stretching (amide I), and at 1560 cm^−1^ (amide II), associated with N-H bending and C-N stretching. The addition of poly(dopamine), which contains catechol groups, introduces characteristic O-H bands in the 3200–3500 cm^−1^ range and bands associated with C=C and C-O stretching around 1500–1600 cm^−1^. Poly(dopamine) interacts with chitosan through hydrogen bonds and other non-covalent interactions. The band at 1400 cm^−1^ is attributed to COO^−^ stretching modes from the indole ring.

The FT-IR spectrum of chitosan cross-linked with glutamic and aspartic acids and poly(dopamine) shows broad bands in the 3200–3500 cm^−1^ range, which may shift or intensify, indicating the presence and interaction of O-H and N-H groups. Changes in band intensity within the 2800–2900 cm^−1^ range may suggest interactions between the aliphatic C-H groups of chitosan and poly(dopamine). Shifts in the band at 1650 cm^−1^ (amide I) indicate the formation of new amide bonds due to cross-linking with the acids, and the appearance of bands in the 1700–1750 cm^−1^ range suggests the presence of carboxyl groups from the acids and the potential formation of ester bonds. The characteristic poly(dopamine) bands in the 1500–1600 cm^−1^ range suggest its integration within the polymeric matrix.

Figure 4 presents the FT-IR spectra of fungal chitosan-based samples modified with conductive nanoparticles and CBD. Cannabidiol exhibits characteristic bands around 3580 cm^−1^ and 3460 cm^−1^, attributed to OH groups. Bands with maxima at 2923 cm^−1^ and 2885 cm^−1^ originate from aliphatic groups (-CH_3_ and -CH_2_-). The bands with maxima at 1573, 1574, and 1577 cm^−1^ are due to C=C stretching vibrations and -CH_2_- bending between 1300 and 1400 cm^−1^. Chitosan shows two overlapping bands (O-H and N-H stretching) near 3400 cm^−1^. Bands corresponding to -CH_3_ and -CH_2_- groups appear at 2923, 2885, and 2879 cm^−1^. The amide I band appears at 1651 cm^−1^, and a band characteristic of the glucopyranose ring is visible at 896 cm^−1^.

The spectrum of fungal chitosan cross-linked with l-Aspartic acid and l-Glutamic acid reveals notable changes, including increased intensity of amide bands, indicating covalent bond formation between the -COOH functional groups of amino acids and the free -NH_2_ groups of the biopolymer (1500–1600 cm^−1^). No bands for ester bonds are present, and the band for free hydroxyl groups remains visible. The cross-linked structure also retains free carboxyl groups from partially incorporated amino acids, confirmed by a band with a maximum at 3200–3300 cm^−1^.

Figure 5 presents the final series of samples modified with poly(dopamine) and Pt/PVP nanoparticles (NPs). In the first band, between 3200 and 3500 cm^−1^, stretching of O-H and N-H groups, characteristic of chitosan, is observed. Following cross-linking, the broadening or change in intensity of these bands may suggest the formation of new hydrogen bonds between the hydroxyl groups of chitosan and the carboxyl groups of the acids. In the 2800–3000 cm^−1^ range, bands associated with the stretching of C-H groups from the aliphatic chains of chitosan are present. Variations in the intensity of these bands could indicate interactions between the chitosan chains and the introduced acid groups.

The amide I band at 1654 cm^−1^ corresponds to the C=O stretching of amide groups, while the amide II band at 1559 cm^−1^ corresponds to N-H deformation and C-N stretching. Post-crosslinking, shifts or changes in these bands’ intensities may signify the formation of new amide bonds or interactions with the carboxyl groups of acids. Additionally, in the 1500–1600 cm^−1^ range, typical of poly(dopamine), new bands associated with catechol groups, as well as bands linked to C=C and C-O stretching, are expected. These additional bands confirm the presence of poly(dopamine) on the chitosan surface.

### 2.3. Swelling Properties Study

Swelling of chitosan-based 3D biomaterials is a key functionality in various biomedical applications such as tissue engineering, drug delivery, and wound dressings. It is insoluble in water and it can swell upon contact, expanding the scaffold’s volume. This process involves water absorption through hydrogen bonding with hydrophilic groups in chitosan, such as amino (-NH_2_) and hydroxyl (-OH) groups. This interaction leads to partial separation of polymer chains and an increase in scaffold volume. The degree of chitosan’s deacetylation and its molecular weight primarily control this swelling behavior. Higher deacetylation, meaning more amino groups, enhances swelling capacity by providing more binding sites for water molecules. Conversely, chitosan with a higher molecular weight forms more compact structures, which may reduce swelling but increase mechanical strength. Swellable chitosan scaffolds are utilized as matrices for cell culture, supporting tissue regeneration by creating a conducive microenvironment. In drug delivery systems, chitosan’s swelling properties enable controlled release of active ingredients. Additionally, chitosan-based wound dressings leverage swelling to absorb wound exudates and maintain a moist healing environment.

Evaluating chitosan-based scaffolds in simulated body fluid (SBF) is essential for assessing their regenerative and tissue-engineering suitability. This process serves as a preliminary biocompatibility assessment for the material, crucial for its safe application as tissue scaffolds or implants. SBF swelling testing mimics biological conditions, as SBF closely resembles body fluids, providing insight into scaffold behavior in realistic biological environments. Swelling in SBF also helps researchers to observe the influence of scaffold properties on cell adhesion, proliferation, and differentiation.

**Figure 6:**
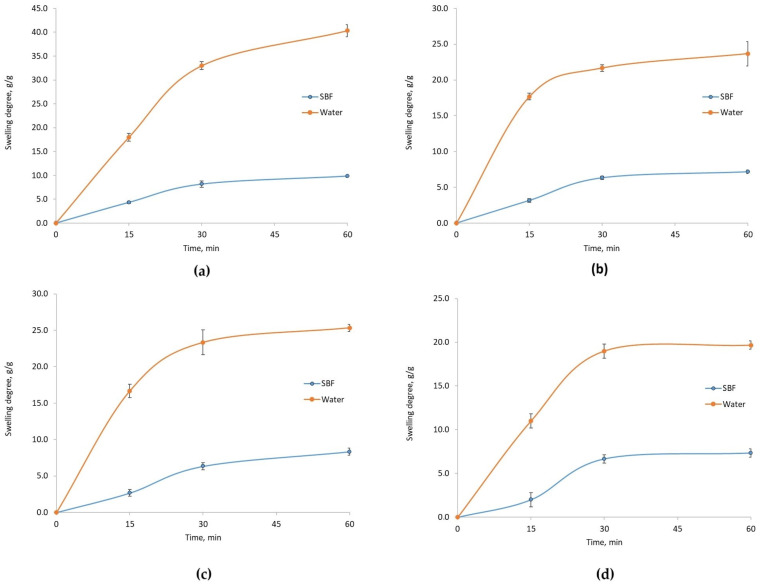
Swlling degree in water and SBF of chitosan biomaterials modified with poly(dopamine)@Au/PVP NPs: (**a**) sample **1**; (**b**) sample **2**; (**c**) sample **3**; (**d**) sample **4**.

**Figure 7:**
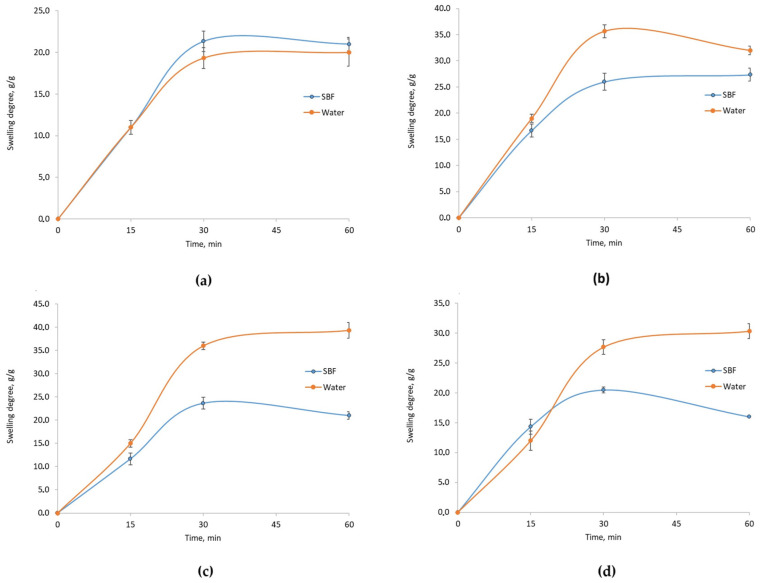
Swelling degree in water and SBF of chitosan biomaterials modified with CBD/Au/PVP NPs: (**a**) sample **5**; (**b**) sample **6**; (**c**) sample **7**; (**d**) sample **8**.

**Figure 8:**
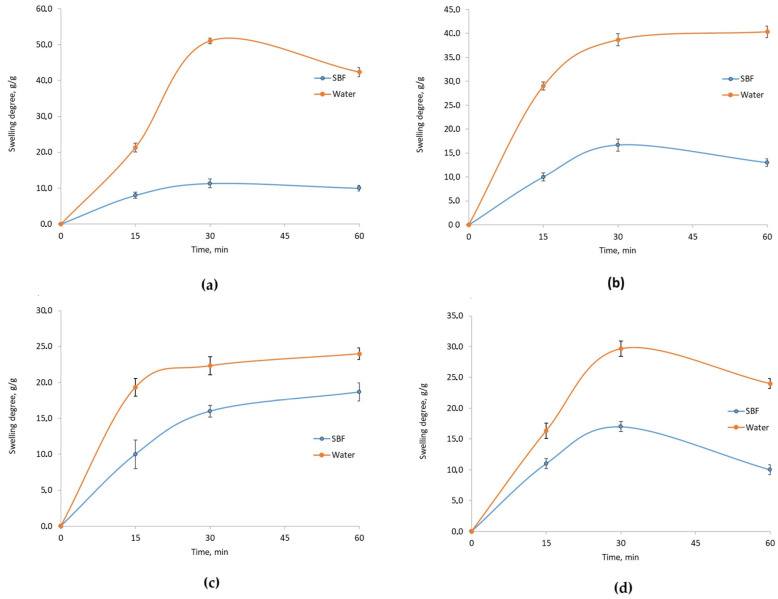
Swelling degree in water and SBF of chitosan biomaterials modified with poly(dopamine)/Pt/PVP NPs: (**a**) sample **9**; (**b**) sample **10**; (**c**) sample **11**; (**d**) sample **12**.

Figure 6, Figure 7 and Figure 8 display the results of swelling studies conducted in different media. Figure 6 shows the mass change in samples **1**–**4** when exposed to water and SBF. A noticeable difference is observed between the Asp 0.84:0 sample and other samples. Asp 0.84:0 exhibited the highest mass increase relative to its initial mass, suggesting favorable swelling properties of the cell scaffold in water. Samples Asp 0.5:0.5, 0.7:0.3, and 0.3:0.7 demonstrated similar mass increases, though no further increase was observed after 30 min, indicating a plateau in swelling. This lack of continued swelling could be problematic for applications where scaffold expansion is necessary to provide cells with adequate growth space.

Conversely, in SBF, the Asp 0.84:0 sample showed the greatest mass and volume gain, suggesting optimal structural properties. The mass continued to increase beyond the 30-min mark. In contrast, samples Asp 0.7:0.3 and 0.3:0.7 displayed suboptimal swelling in SBF, lacking the desired expansion for intended applications.

The results for samples containing CBD additive showed an opposite trend. In Figure 7, the Asp 0.7:0.3 sample displayed the highest mass increase, while the Asp 0.84:0 sample exhibited the lowest. After 30 min, both Asp 0.5:0.5 and Asp 0.7:0.3 showed similar mass increases; however, the Asp 0.5:0.5 sample experienced a subsequent decrease in mass, indicating the onset of material degradation. Early degradation could impair the scaffold’s ability to maintain cell culture structure and reflects a structural instability that leads to premature disintegration in water.

Figure 7 (blue line) also shows mass changes for Asp samples with CBD in contact with SBF. The Asp 0.5:0.5 sample achieved the highest mass increase, stabilizing after 30 min, which could support cell culture by preventing scaffold compression and ensuring structural integrity. However, limited expansion may restrict cell proliferation due to spatial constraints. Samples with Asp ratios of 0.7:0.3 and 0.3:0.7 exhibited early signs of degradation after 30 min, evidenced by a sudden drop in mass, which indicates structural breakdown. 

Figure 8 presents the swelling test results for samples **9**–**12**. The Asp 0.84:0 sample showed the highest mass increase; however, a mass decrease occurred after 30 min, indicating the onset of degradation and structural disintegration. The Asp 0.7:0.3 and 0.3:0.7 samples exhibited the lowest degree of swelling, along with a notable mass loss halfway through the test. The Asp 0.5:0.5 sample maintained its mass after 30 min, indicating a stable degree of swelling with no initiation of structural degradation in water.

When SBF was used as the swelling medium, none of the samples showed sufficient mass gain, with degradation beginning after 30 min. This premature degradation could inhibit cell proliferation. The Asp 0.7:0.3 sample was the only one that did not lose mass halfway through the test, suggesting that it reached its absorption limit and could not further increase in volume.

### 2.4. Degradation and Biodegradation Study

To assess the behavior of newly developed biomaterials for nervous tissue regeneration under conditions similar to the human body, chitosan-based scaffold biodegradation was analyzed in pure simulated body fluid (SBF, blue), SBF with lysozyme (orange), and SBF with collagenase (gray). This evaluation is crucial to understanding the material’s interaction with ions and other bodily components, confirming its biocompatibility and safety. The study identifies enzymatic and hydrolytic degradation mechanisms and the resulting products, which are essential to predict scaffold behavior in biological environments.

Monitoring scaffold degradation is necessary to optimize the biodegradation rate, ensuring sufficient support for regenerating tissue throughout the healing process. It also helps determine how modifications affect scaffold biodegradability, structure, and mechanical properties during degradation. Evaluating mechanical stability in SBF allows us to determine the duration within which scaffolds retain their properties before full degradation, a critical factor for their use in regenerative medicine. While SBF studies do not perfectly mimic biological conditions, they provide valuable preliminary data for in vivo study planning. Identifying degradation products is also important, as some may be toxic or influence tissue regeneration, affecting both the safety of these products and the material’s suitability for tissue engineering.

Studying biodegradation in the presence of lysozyme, a naturally occurring enzyme, has broad implications. Lysozyme, which is key to the body’s defense by breaking down bacterial cell walls, provides a baseline for scaffold biodegradation. Understanding the biodegradation rate in lysozyme-containing environments helps refine chitosan-based materials used in wound dressings and medical applications. This information can lead to improved biomaterials that interact more effectively with the body. Moreover, understanding lysozyme-mediated biodegradation is crucial for drug release control, as chitosan serves as a biodegradable carrier in drug delivery. Knowledge of degradation rates can enhance the control of drug release from scaffolds, potentially improving therapeutic outcomes. Overall, studying biodegradation in lysozyme enriches our understanding of biopolymer degradation processes, guiding the development of medical and environmentally sustainable materials.

**Figure 9:**
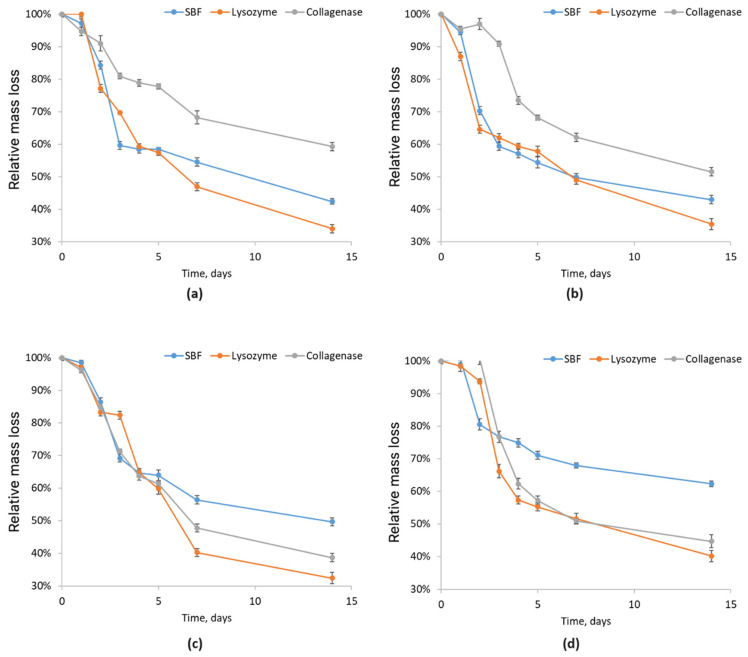
Relative mass loss of chitosan biomaterials modified with poly(dopamine)@Au/PVP NPs: (**a**) sample **1**; (**b**) sample **2**; (**c**) sample **3**; (**d**) sample **4**.

The results of the (bio)degradability analysis for samples 1–4 (chitosan scaffolds modified with poly(dopamine)@Au/PVP NPs) are shown in Figure 9. All samples initially absorbed SBF, leading to swelling, with the Asp 0.84:0 sample reaching the highest mass increase (0.8 g/g) after 24 h. After reaching this peak, all samples gradually decreased in mass over the remaining study period (144 h), marking the start of scaffold degradation. The Asp 0.84:0 sample (blue line) exhibited the slowest mass loss, indicating strong resistance to biodegradation in SBF, while the Asp 0.3:0.7 sample (purple) degraded fastest, showing the greatest susceptibility to SBF degradation. During enzymatic degradation (lysozyme), the Asp 0.84:0 sample mass initially increased, then stabilized after 48 h, and began degrading after 96 h, indicating structural breakdown. The Asp 0.5:0.5, 0.7:0.3, and 0.3:0.7 samples showed similar trends, with a slight mass loss after 96 h, suggesting scaffold stability in the presence of lysozyme.

The degradation study in collagenase aimed to develop scaffolds resistant to enzymatic degradation in tissues. Collagenase, which degrades collagen, plays a significant role in extracellular matrix breakdown, impacting scaffold stability. Understanding its effects enables the creation of more stable materials, enhancing tissue engineering therapies. Results for samples with collagenase (gray line) showed that the Asp 0.84:0 sample absorbed collagenase most effectively but started to degrade after 24 h. The Asp 0.7:0.3 and 0.3:0.7 samples followed similar absorption trends, but Asp 0.7:0.3 degraded more slowly. The Asp 0.5:0.5 sample had the lowest mass gain after 24 h, with very slow degradation, indicating high collagenase resistance.

**Figure 10:**
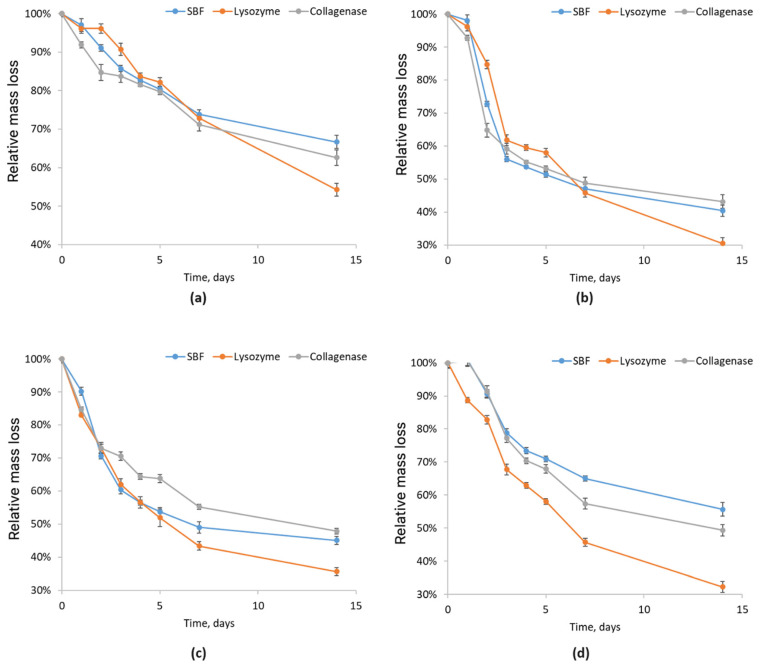
Relative mass loss of chitosan biomaterials modified with CBD/Au/PVP NPs: (**a**) sample **5**; (**b**) sample **6**; (**c**) sample **7**; (**d**) sample **8**.

Figure 10 presents the degradation test results for chitosan biomaterials modified with CBD/Au/PVP nanoparticles in simulated body fluid (SBF, blue), lysozyme (orange), and collagenase (gray) over a period of 144 h. The Asp 0.5:0.5 and 0.7:0.3 samples showed the greatest initial mass increase, while the Asp 0.84:0 sample exhibited the smallest increase. After the first 24 h, all samples showed a reduction in mass. The Asp 0.5:0.5 and 0.7:0.3 samples experienced the largest mass losses, while the Asp 0.84:0 sample had a very gradual and minor decrease, indicating high resistance to degradation in SBF. After 96 h, there was no significant change in the mass loss of the samples, indicating that the samples containing CBD ceased further degradation beyond this point.

In the lysozyme-based degradation study (orange), the Asp 0.3:0.7 sample absorbed the most solution, resulting in the highest initial mass gain, while the Asp 0.7:0.3 sample showed the smallest mass increase. The Asp 0.84:0 sample displayed a very slow mass loss, suggesting low susceptibility to enzymatic degradation by lysozyme. The other samples experienced an initial mass loss of about 0.1 g within the first 24 h, and thereafter showed only a minimal reduction in mass, stabilizing after 96 h.

In the tests with collagenase (gray), the Asp 0.5:0.5 sample demonstrated the best properties, with the highest solution absorption and subsequent volume increase. Degradation began after 24 h, with an approximate mass loss of 0.2 g. The other samples also showed initial absorption within the first 24 h, but their mass remained nearly unchanged after 48 h, indicating strong resistance to collagenase and limited susceptibility to further degradation.

**Figure 11:**
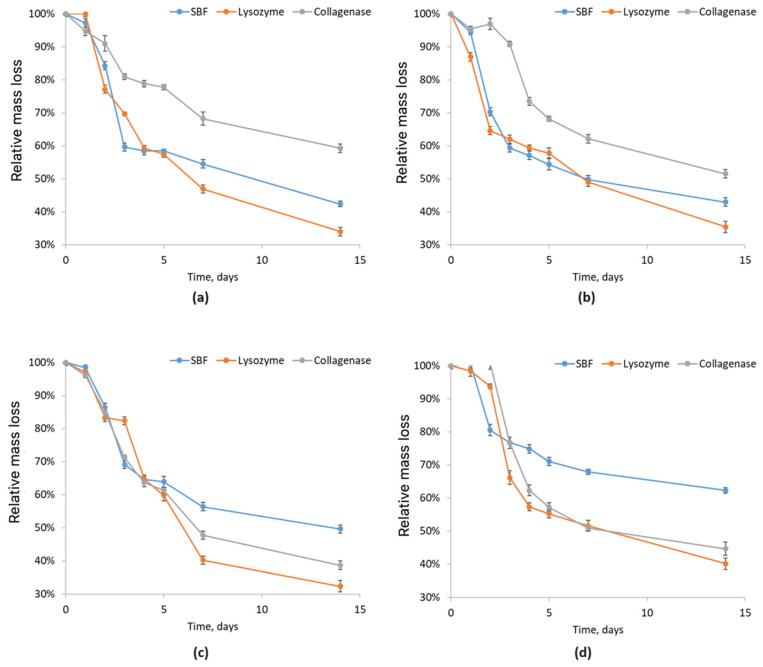
Relative mass loss of chitosan biomaterials modified with poly(dopamine)/Pt/PVP NPs: (**a**) sample **9**; (**b**) sample **10**; (**c**) sample **11**; (**d**) sample **12**.

The study on chitosan biomaterials modified with poly(dopamine)/Pt/PVP nanoparticles in simulated body fluid (SBF) is presented in Figure 11. The Asp 0.84:0 sample exhibited the largest mass increase, while the Asp 0.7:0.3 sample showed the smallest. Notable mass increases were observed within the first 24 h. After this period, a decrease in mass indicated the initiation of the scaffold degradation process. The Asp 0.5:0.5 sample demonstrated the most significant mass loss after 24 h, while the Asp 0.84:0 sample showed the largest mass loss after 48 h. The change in mass stabilized after 96 h, suggesting a possible cessation of structural degradation.

In the lysozyme-induced biodegradation study, all samples absorbed the enzyme solution within the first 24 h of testing. The Asp 0.3:0.7 sample recorded the largest mass increase. Degradation began to occur after 48 h. The Asp 0.5:0.5 and Asp 0.84:0 samples exhibited similar absorption trends, but the Asp 0.84:0 sample showed a faster mass loss, indicating more rapid biodegradation. The most resistant sample to degradation in the presence of the human enzyme lysozyme was the Asp 0.7:0.3 sample, which displayed the smallest mass change throughout the study.

The mass changes in the Asp samples after contact with SBF containing collagenase revealed interesting results for the Asp 0.84:0 and Asp 0.7:0.3 samples. The Asp 0.84:0 sample absorbed the collagenase solution for 48 h before losing 0.3 g, whereas the Asp 0.7:0.3 sample lost the same amount of mass after only 24 h. The degradation of the Asp 0.84:0 sample occurred during the third day of the study. The Asp 0.5:0.5 and Asp 0.3:0.7 samples absorbed the solution for 48 h and subsequently degraded at a slow rate.

### 2.5. Cytotoxicity Study

The cytotoxicity study of chitosan-based cell scaffolds, conducted on astrocyte cells, aims to assess the safety of this material in a biomedical context and to identify potential benefits and risks associated with its use. Chitosan is a promising material in biomedicine due to its biocompatibility and biodegradability; however, a cytotoxicity study is necessary to evaluate its effect on astrocyte cells. Cell viability was first assessed through microscopic observations. Analyzing the obtained results will help determine whether chitosan and its additives affect astrocyte cell survival and identify optimal conditions for their use as cell scaffolds.

This test can also help identify potential side effects of chitosan on astrocyte cells and optimize the conditions for its application. Positive results could provide a foundation for further studies on the use of chitosan in treating neurological diseases, such as in tissue engineering or brain regeneration therapy. Overall, the aim of the study is to determine whether chemically crosslinked chitosan modified with PDA/CBD and conductive nanoparticles can be safely used as a cellular scaffold material for astrocyte cells.

**Figure 12:**
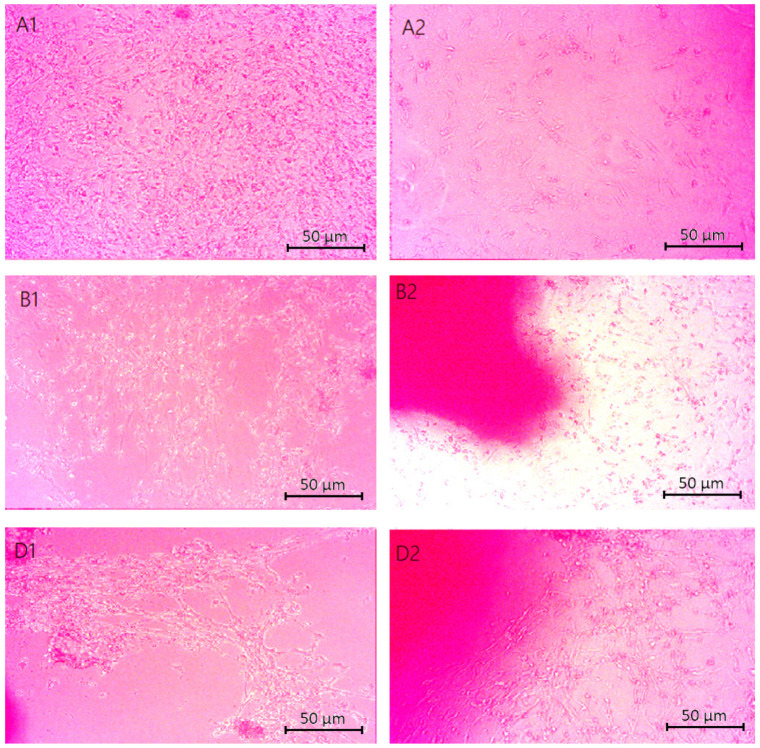
Microscopic images (40× magnification) of chitosan biomaterials modified with poly(dopamine) NPs cytotoxicity results performed on nerve cells (astrocytes): (**A1**,**A2**) Asp:Glu 0.84:0), (**B1**,**B2**) Asp:Glu 0.5:0.5 (**D1**,**D2**) Asp:Glu 0.3:0.7.

Figure 12 shows microscopic images of chitosan biomaterials modified with poly(dopamine)@Au/PVP nanoparticles in direct contact with astrocyte cells. Characteristic properties of live and properly developing cells include a spindle or elongated shape. The scaffolds subjected to the study did not show toxicity towards the cells, as evidenced by the formation of monolayers from astrocytes or their clustering around the samples. The only sample that exhibited cytotoxicity was the one containing a ratio of Asp acid 0.7:0.3.

**Figure 13:**
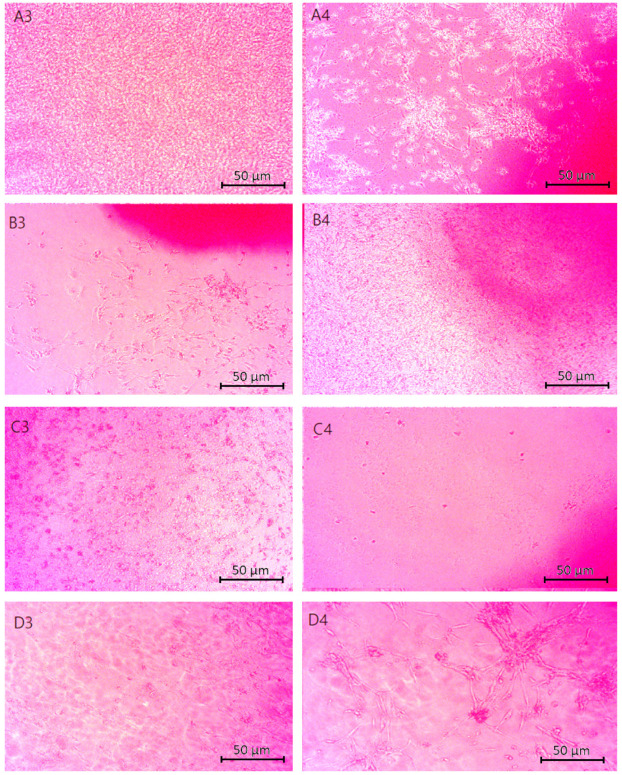
Microscopic images (40× magnification) of chitosan biomaterials modified CBD cytotoxicity results performed on nerve cells (astrocytes): (**A3**,**A4**) Asp:Glu 0.84:0, (**B3**,**B4**) Asp:Glu 0.5:0.5, (**C3**,**C4**) Asp:Glu 0.7:0.3, (**D3**,**D4**) Asp:Glu 0.3:0.7.

Figure 13 displays the cytotoxicity results for cells in the vicinity of samples containing CBD. The photographs reveal developing cells that did not exhibit signs of apoptosis upon contact with the samples. The cells are interconnected, forming a monolayer. The pink spots visible in the images represent the biomaterial. These results indicate a lack of contact inhibition in cell growth.

**Figure 14:**
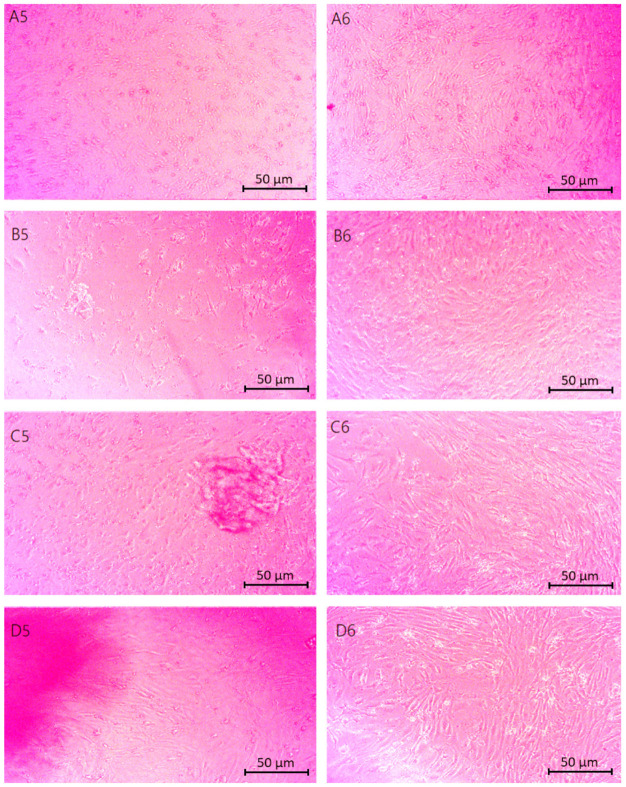
Microscopic images (40× magnification) of biomaterials modified with poly(dopamine) cytotoxicity results performed on neural cells (astrocytes): (**A5**,**A6**) Asp:Glu 0.84:0, (**B5**,**B6**) Asp:Glu 0.5:0.5, (**C5**,**C6**) Asp:Glu 0.7:0.3, (**D5**,**D6**) Asp:Glu 0.3:0.7.

The cytotoxicity assessment of the cellular scaffold samples containing poly(dopamine) shown in Figure 14 indicated no negative effects on the viability or proliferation of astrocyte cells. The cells displayed spindle-shaped morphologies, suggesting undisturbed development.

To further investigate newly developed biomaterials, a quantitative XTT assay has been carried out. The XTT test is a commonly used method for assessing cell viability and metabolism, based on the activity of mitochondrial dehydrogenases that reduce tetrazolium salt (XTT) to water-soluble formazan, and is recommended by ISO 10993-5 norm. The XTT test was performed on the astrocytoma cell line 1321N1 in the context of testing chitosan biomaterials enriched with cannabidiol (CBD), polydopamine (PDA), and gold/platinum nanoparticles coated with polyvinylpyrrolidone (PVP), to determine the effect of these materials on the viability of astrocytoma cells.

Chitosan, as a biocompatible and biodegradable polymer, has extensive applications in tissue engineering and drug delivery systems. However, its chemical modification can impact the biological interactions between cells and biomaterials. The incorporation of CBD may influence the proliferation of nervous cells due to its anti-inflammatory and antioxidant properties. Poly(dopamine), known for enhancing surface modification and improving cell adhesion, can strengthen interactions between biomaterials and cells, which is vital for the bioactivity and stability of the material. Additionally, gold nanoparticles (AuNP) and platinum nanoparticles (PtNP) coated with PVP, recognized for their catalytic and photothermal properties, can modulate cellular responses through molecular and photobiological interactions, thereby positively influencing cell proliferation, migration, and maturation.

**Figure 15:**
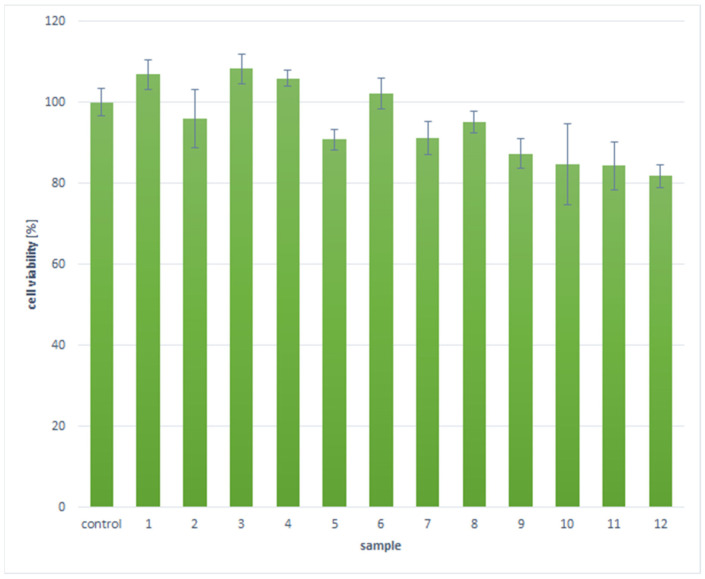
XTT assay results of the newly developed biomaterials.

The XTT test conducted in this research allowed for the evaluation of cytotoxicity, potential biocompatibility, and the effectiveness of the biomaterial in interaction with astrocytoma cells, which is essential for developing nerve guidance conduits capable of electrically stimulating tissue regeneration. As shown in Figure 15, all evaluated samples exhibited no cytotoxicity, as cell viability was above 70%, which meets the ISO 10993-5 standard for non-toxicity. However, significant differences in biological activity were observed among the different samples, attributed to variations in chemical composition and the degree of deacetylation of chitosan used in synthesizing the biomaterials.

The poorest results were noted for PtNPs coated with PVP, as cell viability was lower compared to astrocytes cultured under standard conditions. Conversely, samples 1, 3, 4, and 6 demonstrated a higher number of metabolically active cells compared to the control. In most cases, the least favorable outcomes were associated with chitosan exhibiting a 90% deacetylation degree. The quantitative cytotoxicity assay results correlate well with the qualitative analysis of cell morphology.

### 2.6. Biomaterials Morphology Study

The appropriate morphology of materials intended for use as potential cell scaffolds is crucial for several reasons. A highly porous structure is essential, as many biologically inert and biocompatible polymeric devices are available for drug delivery or cell culturing. However, due to their very low porosity or solid nature, these materials cannot fulfill the necessary requirements for these applications.

**Figure 16:**
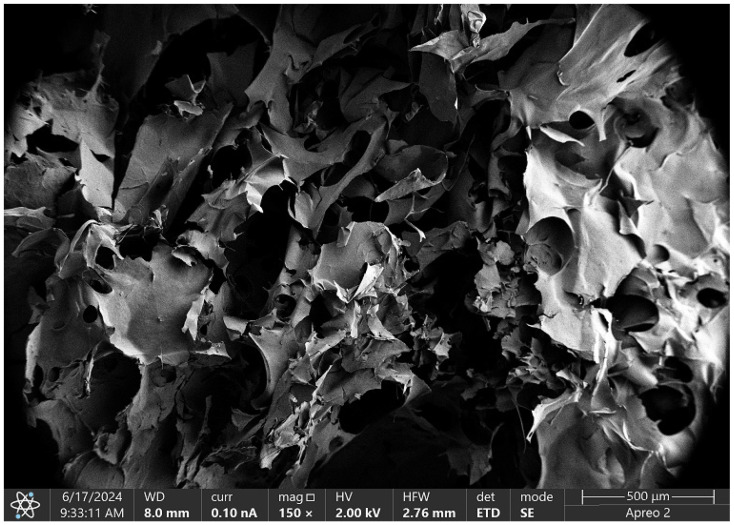
SEM micrograph of the Asp:Glu 0.84:0 + poly(dopamine) sample.

The microscopic image in Figure 16 illustrates the structure of the Asp 0.84:0 sample modified with poly(dopamine). Consistent with previous samples, this scaffold exhibits a significant number of pores and a rough texture. The poly(dopamine) is evenly deposited on the scaffold surface, appearing as darker, thinner spots in the SEM image. This even distribution of poly(dopamine), combined with conductive nanoparticles, enhances the biocompatibility of the scaffold, facilitating cell adhesion and potentially serving as a primer for the deposition of other biomolecules.

**Figure 17:**
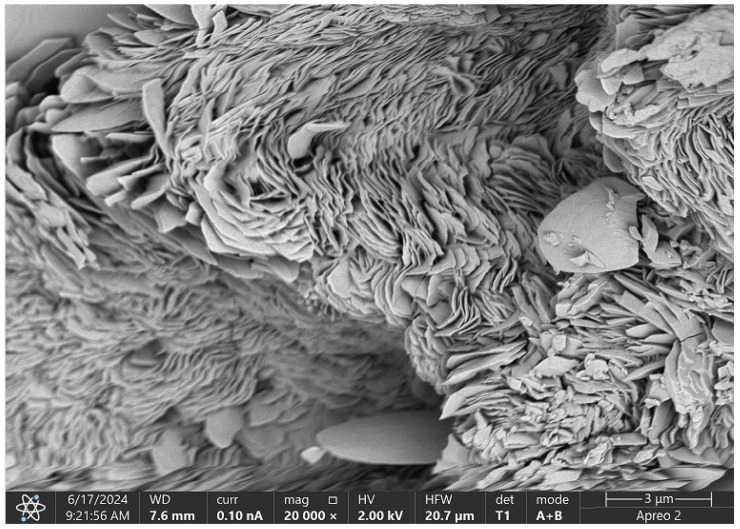
SEM micrograph of the Asp:Glu 0.84:0 + CBD sample.

Figure 17 presents the SEM microscopic image of the Asp 0.84:0 sample with the addition of CBD. This three-dimensional scaffold exhibits optimal structural properties for tissue engineering applications. The highly magnified image reveals a complex, layered, and fibrous structure characterized by fine textures and small-scale features, indicating a high level of surface roughness and intricate morphology.

The scaffold comprises numerous thin, sheet-like layers arranged in an irregular fashion, creating a network of fine chitosan fibers that forms a complex three-dimensional architecture. The incorporation of CBD not only enhances the flexibility and strength of the scaffold but also contributes to its overall functionality. The rough surface of the scaffold promotes cell adhesion, proliferation, and differentiation, essential for effective tissue engineering.

Additionally, the uniform microstructure ensures even distribution of CBD, which possesses anti-inflammatory and antioxidant properties. This can further facilitate tissue regeneration while helping to reduce inflammation, making the scaffold a promising candidate for applications in regenerative medicine.

**Figure 18:**
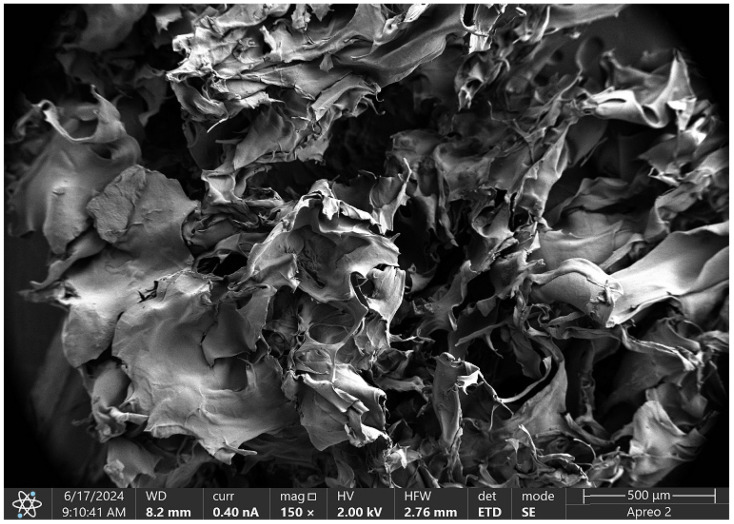
SEM micrograph of the Asp:Glu 0.3:0.7 poly(dopamine) sample.

The three-dimensional cellular scaffold based on chitosan, enhanced with poly(dopamine), is depicted in Figure 18. This scaffold is characterized by numerous irregular pores that facilitate nutrient diffusion and cell migration. The structure comprises small fibers that form a complex spatial network, significantly increasing the surface area available for cell adhesion.

The rough surface texture of the scaffold promotes cell–cell interactions, which is crucial for effective tissue formation. Additionally, the microstructure features intricate details, such as wrinkles, that further enhance the active surface area of the scaffold. With an image scale of 500 μm, it is evident that the observed structures are in the micrometric range, a characteristic typical of scaffolds utilized in tissue engineering. This design creates an optimal environment for cell culture and differentiation, making it a suitable candidate for regenerative medicine applications.

## 3. Discussion

**Figure 19:**
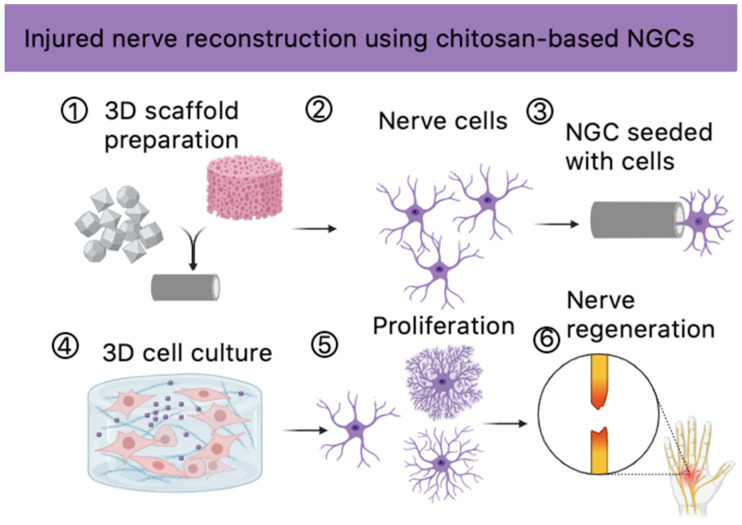
Potential future application of the newly developed biomaterials (created via Biorender).

The aim of this work was to develop and characterize three-dimensional chitosan-based cellular scaffolds for nervous tissue regeneration (Figure 19). The research methods demonstrated that scaffolds synthesized from chitosan, l-Aspartic acid, l-Glutamic acid, propylene glycol, and active additives such as poly(dopamine) and CBD exhibited several beneficial features, confirming their potential for use in tissue engineering aimed at nervous tissue regeneration.

FT-IR spectroscopy revealed molecular interactions between chitosan and the amino acids. The observed shifts and changes in band intensities after cross-linking indicated the formation of new amide bonds and interactions with the carboxyl groups of the acids. Notably, the presence of new bands associated with catechol groups and C=C and C-O stretching confirmed the successful incorporation of poly(dopamine) onto the chitosan surface. Additionally, samples with CBD displayed characteristic bands around 3580 cm^−1^ from OH groups. These findings indicated that adding active compounds to the scaffold matrices did not pose a risk of generating toxic byproducts for cells.

A critical aspect of this research involved assessing the samples’ swelling capacity in environments similar to those found in the human body. Swelling studies conducted in water, SBF, and DMEM medium indicated that the addition of poly(dopamine) and CBD significantly impacted the scaffolds’ swelling properties and structural stability. The Asp 0.84:0 sample with spotted poly(dopamine) demonstrated the highest mass increase, indicating superior swelling properties, which may facilitate cell growth in applications requiring substantial space.

However, the rapid degradation of samples, especially those containing CBD, highlights the need for further optimization of their composition and preparation methods to ensure structural stability over time. The Asp 0.5:0.5 sample, containing poly(dopamine), exhibited the best mass stability in water, whereas the Asp 0.84:0 sample showed optimal swelling in SBF, positively influencing cell culture outcomes. The Asp 0.5:0.5 sample with CBD also exhibited notable swelling but did not sustain further mass increases after 30 min, potentially limiting its utility for cell cultures due to insufficient surface area for cell development. In DMEM medium, the Asp 0.3:0.7 sample with poly(dopamine) displayed the best swelling properties, while the Asp 0.7:0.3 sample with CBD, despite a significant mass increase, began degrading prematurely.

The biodegradation tests aimed to examine scaffold behavior under conditions mimicking those in the human body, confirming biocompatibility and safety. These studies revealed that bond hydrolysis reactions occurred due to enzymes or solutions simulating the body’s environment. The Asp 0.84:0 samples, regardless of modification, demonstrated the highest stability and resistance to biodegradation in SBF, suggesting their potential for long-term mechanical support during tissue regeneration. Conversely, the Asp 0.5:0.5 and Asp 0.7:0.3 samples, particularly those containing CBD, exhibited faster degradation, which may be advantageous in scenarios requiring shorter support durations. In lysozyme conditions, the Asp 0.84:0 sample with spotted poly(dopamine) exhibited the most significant mass increase and provided the largest area for cell colonization, with degradation commencing only after 96 h, indicating extended stability in lysozyme.

Cytotoxicity assessments of the scaffolds indicated that nearly all samples, both with poly(dopamine) and CBD, were biocompatible, demonstrating no negative impact on cell viability. The Asp 0.84:0 sample with CBD stood out as the best, promoting the healthy development of astrocyte cells, characterized by significant cell proliferation and monolayer formation. The XTT quantitative cytotoxicity assay revealed that gold nanoparticles enhanced astrocyte proliferation, likely by influencing the cell cycle, while platinum nanoparticles had a mild negative effect [43]. Appropriate chemical compositions were shown to enhance cell proliferation, potentially accelerating nerve tissue regeneration. Additionally, the conductive nature of the biomaterials allows for electric impulse transmission and external stimulation via electric fields. 

SEM micrograph analysis confirmed that all analyzed samples, whether containing poly(dopamine) or CBD, exhibited high porosity and roughness, which foster cell adhesion and proliferation while facilitating nutrient diffusion. The incorporation of poly(dopamine) and CBD not only improved biocompatibility but also enhanced scaffold functionality; poly(dopamine) promoted cell adhesion, while CBD’s anti-inflammatory properties supported tissue regeneration.

## 4. Materials and Methods

### 4.1. Materials

For the experiments, fungal chitosan 100–300 cps (890,000 avg. g/mol molar mass) obtained from *Aspergillus Niger* has been used, purchased from PolAura, Dywity, Poland, with deacetylation degree DD = 88%. The choice of non-animal-derived raw biopolymer was based on our previous experiments, which revealed increased biocompatibility and repeatability than crustaceans-based ones. l-Gspartic and l-Glutamic acids, SBF (simulated body fluid), ethanol, propylene glycol, dopamine hydrochloride, TRIS buffer, cannabidiol, DMEM, trypsin, collagenase derived from *Clostridium histolyticum,* and human lysozyme were purchased from SigmaAldrich, Poznań, Poland. XTT assay (Roche) was purchased from SigmaAldrich.

### 4.2. Methods

#### 4.2.1. Transmission Electron Microscopy (TEM)

Metallic nanoparticles were prepared by the procedure described in the previous paper [44]. Ready NPs were characterized regarding their morphology by TEM microscope. For this purpose, NPs solutions were instilled onto Cu meshes, dried, and placed in a microscope chamber. Photographs were taken using TEM microscope JEOL JEM2100 purchased from (Jeol USA, lnc., Peabody, MA, USA).

#### 4.2.2. Biomaterials Synthesis

Poly(dopamine) was obtained via the self-oxidation process of dopamine chloride in the presence of TRIS buffer. All biomaterials have been prepared in the field of microwave radiation. Their composition is given in Table 1. The first step in the preparation of three-dimensional chitosan-based scaffolds was to prepare twelve samples containing different amounts of components. Each sample was prepared in the same way. 15–25 mL of water was poured into a 250 mL beaker and the appropriate proportions of aspartic and glutamic acid were added. The solution was stirred on a magnetic stirrer at an elevated temperature of approximately 80 °C. After dissolving a specified quantity of amino acids in the water, 0.5 g of 100–300 cpu fungal chitosan was added to each of the twelve samples. Primary studies have shown scaffolds prepared from the chitosan of cps 10–120 after swelling with aqueous media tend to lose their integrity and have insufficient durability. Therefore, for the following study, biopolymer of higher molecular mass was used. Mixing continued until a clear solution was obtained. Four samples with different Asp:Glu contents were supplemented with 5 mL of the poly(dopamine) aqueous solution prepared at that time. The next four samples contained an addition of 25 mL of 5% CBD solution in ethanol, which was further evaporated. Metallic NPs were prepared as described in our previous paper [44].

Then, using an automatic pipette, after 10 min, 10 mL of propylene glycol was added to the mixture. The prepared samples in beakers were placed in a microwave reactor for about 3 min at P = 800 W. The cross-linked samples were washed 4 times with distilled water until a neutral pH was obtained. The obtained hydrogels were dried on paper towels. The prepared samples were placed in sterile plastic vessels, frozen in a freezer and subsequently freeze-dried for 24 h. The freeze-dried samples were sealed in sterile vessels. Four of the freeze-dried samples containing water, chitosan, Asp:Glu acid, and glycol were sprinkled with a solution of poly(dopamine) in the amount of 4.5 mL for each sample. The sprinkled samples were again freeze-dried for 24 h. After freeze-drying, the samples were also sealed in airtight vessels to prevent contact with moisture.

#### 4.2.3. FT-IR Analysis

The analysis was performed using a Thermo Nicolet Nexus 470 spectrometer (Thermo Fisher Scientific, Waltham, MA, USA) equipped with an ATR attachment and a diamond/ZnSe crystal. The studies were performed using the spectral range of 4000–500 cm^−1^.

#### 4.2.4. Swelling Properties Study

Swelling capacity is the amount of solvent absorbed by a given substance in a given time per mass unit (e.g., g/g). In this study, three solutions were used to verify the biomaterials’ behaviors in different media. For each type of solution, 3 repetitions were made. Three sterile vessels containing swelling solutions were prepared (distilled water, SBF, and DMEM medium 4.5 g/L with l-Glutamine). Three samples from each of the twelve three-dimensional scaffolds were prepared, weighing approximately 0.010 g and immersed in the wells of the plate with a given solution. The measurement consisted of observing changes in the mass of samples immersed in the appropriate solution at specific time intervals for 60 min. The first measurement was carried out after 30 min, the second after 60 min. The mass was measured on a Radwag PS 1000/C/1 analytical balance with an accuracy of 0.001 g.

#### 4.2.5. Degradation and Biodegradation Study

Biodegradation analysis is the measurement of the mass change in a given sample exposed to a specific solution (medium or enzyme). In three closed plates, 5 mL of solutions were prepared, namely SBF, collagenase solution from *Clostridium histolyticum* in SBF (2 mg/200 mL), and Human lysozyme solution in SBF (2 mg/200 mL). Three samples were prepared from each of the twelve three-dimensional scaffolds weighing approximately 0.010 g and immersed in the wells of the plate with dedicated solution. During the measurement mass changes in samples immersed in the appropriate solution at specific time intervals for 144 h were determined. The first measurement was performed after 24 h. The experiment was performed using Radwag PS 1000/C/1 analytical balance with an accuracy of 0.001 g.

#### 4.2.6. Cytotoxicity Study

The cytotoxicity study began with the culture of astrocyte cells of the 1321N1 cell line. The cell culture medium used for the experiments was composed of DMEM + 2 mM Glutamine + 10% Fetal Bovine Serum (FBS) and 1% antibiotic/antimycotic solution at 37 °C, 5% CO_2_ concentration, and high humidity. A total of 12 samples of three-dimensional chitosan-based scaffolds of similar mass were introduced into the wells. After 48 h, microscopic images of each well containing the sample and medium with cells were taken. The study was performed using an inverted optical microscope at 40× magnification with a Delta Optical MET-200-TRF microscope (Planeta Oczu, Zielona Góra, Poland). Quantitative cytotoxicity was carried out on samples modified with nanoparticles via XTT assay carried out according to the producer’s protocol.

#### 4.2.7. Morphology Study

SEM study was performed on 3 selected samples with the most favorable biological properties, i.e., Asp:Glu 0.3:0.7 + poly(dopamine), Asp:Glu 0.84:0 + CBD, and Asp:Glu 0.84:0 spotted poly(dopamine). The analysis was performed using a high-resolution Apreo 2 S LoVac scanning electron microscope (Thermo Fisher Scientific) equipped with X-ray energy dispersive spectrometers—EDS detectors: UltraDry (Thermo Fisher Scientific) and Octane Elect (EDAX Ametek GmbH), Labsoft, Poland.

## Figures and Tables

**Table 1:** Samples composition.

Sample	Chitosan Amount [g]	Asp:Glu [g:g]	Glycol [mL]	H_2_O [mL]	Modifier
1	0.5	0.84:0	10	20	5 mL poly(dopamine)@Au/PVP NPs
2		0.5:0.5			
3		0.7:0.3			
4		0.3:0.7			
5		0.84:0		15	10 mL CBD/Et@Au/PVP NPs
6		0.5:0.5			
7		0.7:0.3			
8		0.3:0.7			
9		0.84:0		25	4.5 mL poly(dopamine)@Pt/PVP NPs
10		0.5:0.5			
11		0.7:0.3			
12		0.3:0.7			

## Data Availability

Data available on request.
